# Speaking Well and Feeling Good: Age-Related Differences in the Affective Language of Resting State Thought

**DOI:** 10.1007/s42761-024-00239-z

**Published:** 2024-06-24

**Authors:** Teodora Stoica, Eric S. Andrews, Austin M. Deffner, Christopher Griffith, Matthew D. Grilli, Jessica R. Andrews-Hanna

**Affiliations:** 1 Department of Psychology, University of Arizona, 1503 E. University Blvd, Tucson, AZ 85721 USA; 2 Cognitive Science, University of Arizona, Tucson, AZ USA; 3 Evelyn F. McKnight Brain Institute, University of Arizona, Tucso, AZ USA; 4 Department of Neurology, University of Arizona, Tucson, AZ USA

**Keywords:** Well-being, Thought, Aging, Emotion, Language

## Abstract

Despite the prevalence and importance of resting state thought for daily functioning and psychological well-being, it remains unclear how such thoughts differ between young and older adults. Age-related differences in the affective tone of resting state thoughts, including the affective language used to describe them, could be a novel manifestation of the positivity effect, with implications for well-being. To examine this possibility, a total of 77 young adults (*M* = 24.9 years, 18–35 years) and 74 cognitively normal older adults (*M* = 68.6 years, 58–83 years) spoke their thoughts freely during a think-aloud paradigm across two studies. The emotional properties of spoken words and participants’ retrospective self-reported affective experiences were computed and examined for age differences and relationships with psychological well-being. Study 1, conducted before the start of the COVID-19 pandemic, revealed that older adults exhibited more diversity of positive, but not negative, affectively tinged words compared to young adults and more positive self-reported thoughts. Despite being conducted virtually during the COVID-19 pandemic, study 2 replicated many of study 1’s findings, generalizing results across samples and study contexts. In an aggregated analysis of both samples, positive diversity predicted higher well-being beyond other metrics of affective tone, and the relationship between positive diversity and well-being was not moderated by age. Considering that older adults also exhibited higher well-being, these results hint at the possibility that cognitively healthy older adults’ propensity to experience more diverse positive concepts during natural periods of restful thought may partly underlie age-related differences in well-being and reveal a novel expression of the positivity effect.

**Supplementary Information:**

The online version contains supplementary material available at 10.1007/s42761-024-00239-z.

Although often viewed through a lens of decline, typical aging is accompanied by emotional and cognitive gains. There is ample evidence, for example, that older adulthood may bring changes in psychological well-being (Reed et al., 2014; Reed & Carstensen, 2012; but see Hansen & Blekesaune, 2022), biases toward positive stimuli (Charles et al., 2003; Grühn & Scheibe, 2008; Mather & Carstensen, 2005; but see Steptoe et al., 2015), and stability or improvement in vocabulary and semantic knowledge (Park & Reuter-Lorenz, 2009; Salthouse, 2009; Shafto & Tyler, 2014; Singh-Manoux et al., 2012). Affective language—the emotional tone underlying the words we use to express our thoughts—lies at the intersection of these emotional and language gains in older adults. Affective language may provide a unique window into the thoughts and emotions that fill much of our day, possibly offering new insights into age-related changes in well-being and cognitive shifts toward positivity.

The age-related “positivity effect” has been robustly documented in studies of emotional visual attention (Isaacowitz et al., 2006; Mather & Carstensen, 2003), working memory (Mikels et al., 2005), short-term memory (Charles et al., 2003), autobiographical memory (Kennedy et al., 2004), and decision making (Löckenhoff & Carstensen, 2008), yet it is unclear whether it persists during naturalistic speech. Some studies have found that with increasing age, individuals use more positive (compared to negative) affective words and use fewer references to the self (Pennebaker & Stone, 2003). Importantly, quantitative evidence links language use to psychosocial variables such as personality and emotional stability (Schwartz et al., 2013), echoing similar work connecting frequent use of positive words to positive emotion states (Kern et al., 2016) in both young (Quoidbach et al., 2014; Rivera-Hernandez et al., 2021) and midlife adults (Urban-Wojcik et al., 2022). According to the socioemotional selectivity theory, a shift toward positive stimuli over the lifespan can be explained by a change in motivation and goals as time horizons become increasingly constrained (Carstensen et al., 1999). As many people approach later stages of life, goals associated with emotional meaning become more salient, whereas goals associated with acquiring knowledge for future use become less essential. Whether the positivity effect endures in the underlying affective language of unprompted thought in older age is not yet known.

The relationship between naturalistic thought and language is a complex and debated topic in the fields of psychology (Carroll, 2012), linguistics (Chomsky, 2000), and cognitive science (Shapiro, 2019). Although no consensus exists on the multifaceted and likely dynamic association between thought and language, there is agreement that one influences the other. Importantly, in our everyday life, thoughts and emotions often arise unprompted during the many so-called resting states that are sprinkled throughout the waking day (Callard et al., 2013). Despite being commonly used as a context for neuroimaging analyses (Buckner et al., 2008; Koch et al., 2016), little research has quantified resting state thoughts as a cognitive-affective construct (e.g., Diaz et al., 2013). Most characterizations of resting state cognition follow from indirect theoretical considerations (Callard & Margulies, 2014) or retrospective characterizations subject to memory biases and metacognitive impairments (Hurlburt & Heavey, 2015) that may be exacerbated in aging.

One tool to overcome prior gaps in measuring unprompted thoughts is the think-aloud paradigm, where participants are asked to speak their thoughts aloud in real time, in an unconstrained manner. First used to explore cognitive processes and the content of consciousness (Ericsson & Simon, 1993; Russo et al., 1989), the think-aloud paradigm has been a useful method for investigating processes spanning language comprehension (Magliano & Millis, 2003), autobiographical memory (Haque & Conway, 2001), confabulation (Burgess & Shallice, 1996), individual differences in personallity (Holleran & Mehl, 2008), trait rumination (Li et al., 2022; Raffaelli et al., 2021), and recently, creativity (Raffaelli et al., 2023). Thus, the think-aloud paradigm can offer a unique window into the content of one’s inner thoughts and emotions through the exploration of its linguistic features. In the present manuscript, we employ the think-aloud paradigm to gain insight into the use and diversity of affective language in older and younger adults, and to explore how such language relates to well-being.

Growing evidence suggests that language helps a person initially acquire and then later support the representations that comprise emotion concept knowledge (Barrett, 2006; Lindquist et al., 2015). Quantifying the affective tone of language during resting-state thought—including the frequency and diversity of affectively toned words—may provide key insights into individual differences in this rich cache of emotional concept knowledge. Correlational findings from neuroimaging studies of emotion indicate that many brain regions typically associated with language, and semantic processing in particular, are also involved during emotion processing (Satpute & Lindquist, 2021). Furthermore, naturalistic vocabularies derived from unprompted contexts may reveal how people wield this emotional concept knowledge in their most frequent thoughts (Boyd & Pennebaker, 2017; Pennebaker & King, 1999; Tausczik & Pennebaker, 2010; Zipf, 2012) and may even influence the construction of adaptive or maladaptive emotional experiences depending on their valence (Vine et al., 2020), such as the tendency to retrieve and repetitively rehearse autobiographical and negatively valenced content (rumination) in major depressive disorder (American Psychiatric Association, 2013).

## Purpose of the Present Studies

Across two samples of participants in different experimental settings, we used a think-aloud paradigm to test our hypothesis that the positivity effect persists in the resting-state thought of older individuals and relates to well-being. In both studies, we audio recorded young and older adults as they spoke aloud their “stream of consciousness” during an unconstrained period of rest. In study 1 (conducted before the COVID-19 pandemic), the think-aloud paradigm was administered in an MRI scanner, while in study 2 (conducted during the height of the COVID-19 pandemic), participants completed the think-aloud paradigm through a virtual platform in the comfort of their own home. Across both studies, we assessed the use of emotional content (also referred to as “affective tone”) by examining the overall frequency of positively and negatively valenced words, as well as the diversity (or breadth) of positive and negative vocabulary in light of research linking emotional diversity to enhanced well-being (Quoidbach et al., 2014; Vine et al., 2020). Motivated by the positivity effect (Mather & Carstensen, 2005; Reed & Carstensen, 2012), we predicted that older adults would demonstrate higher positive tone, but also higher positive diversity (a higher ratio of unique positive words to total positive words) compared to young adults. We further predict that positive tone and positive diversity in older adults will relate to higher psychological well-being, demonstrating novel evidence of the positivity effect in this population.

## Study 1

### Study 1 Method

**Table 1:** Sociodemographic data

		Study 1					Study 2				
			YA (*SD*)	OA (*SD*)	YA > OA Significance test	*p* value		YA (*SD*)	OA (*SD*)	YA > OA Significance test	*p* value
*N*		48	*22*	*26*			**103**	55	48		
% Female			55	81	*W* = 211	.050		74.5	72.9	*W* = 1063	.632
*Age*			27.1 (3.6)	67.6 (5.9)				22 (5.1)	70 (6)		
*Race*	American Indian/Alaskan Native		0	0				1	0		
	Asian		7	1				6	0		
	Black or African American		0	0				0	2		
	Native Hawaiian or Other Pacific Islander		0	0				1	0		
	White		10	24	*W* = 134	.003**		33	46	*W* = 759	.016*
	Not reported		5	1				14	0		
*Ethnicity*	Non-Hispanic		15	26				47	48		
	Hispanic		6	0	*W* = 173	.007**		8	0	*W* = 2,572	< .001**
*Gender*	Male		10	5				14	14		
	Female		12	21				41	34		
	Non-binary		NA	NA				0	0		
	Not listed		NA	NA				0	0		
	Prefer not to answer		NA	NA				0	0		
Education (yrs)			17.8 (1.86)	16.7 (3.2)	*W* = 236	.392		15.18 (2.24)	17.40 (1.84)	*W* = 2,184.5	< .001**
Subjective SES (MacArthur Scale of Subjective Social Status)			NA	NA				6.49 (1.56)	6.34 (1.68)	*W* = 988	.712
Vocabulary (Wechsler Adult Intelligence Scale IV)			NA	NA				45.66 (6.74)	49.09 (4.58)	*W* = 2,320	.014*

Age group differences within each study were evaluated with a Mann–Whitney *U* test for continuous variables. In study 1, gender was categorized in an open-ended way, without any selection options. In study 2, options for gender were “female,” “male,” “non-binary,” “not listed” or “Prefer not to answer”

*NA* not assessed

*p < .05, **p < .01

Study 1 consisted of younger (aged 18–35) and older participants (aged 60–85) recruited from the University of Arizona and the greater Tucson community, prior to the COVID-19 pandemic. Exclusion criteria were current drug abuse, major psychiatric disease apart from mild-to-moderate depression, history of moderate to severe head trauma, neurodevelopmental and neurological disorders, not being fluent in English, and a score lower than 26 on the Mini-Mental State Examination. Nine participants’ audio files were inaudible, resulting in an analyzed sample of 22 young adults (55% female; age range: 22–35 years old; mean age 27.1 years old) and 26 older adults (81% female; age range: 58–82 years old; mean age 67.6 years). See Table 1 for detailed sociodemographic data for both studies. Written informed consent was obtained from all participants, and all procedures were performed in accordance with the relevant guidelines and regulations and approved by the University of Arizona’s Institutional Review Board.

#### Think-Aloud Paradigm

**Fig. 1:**
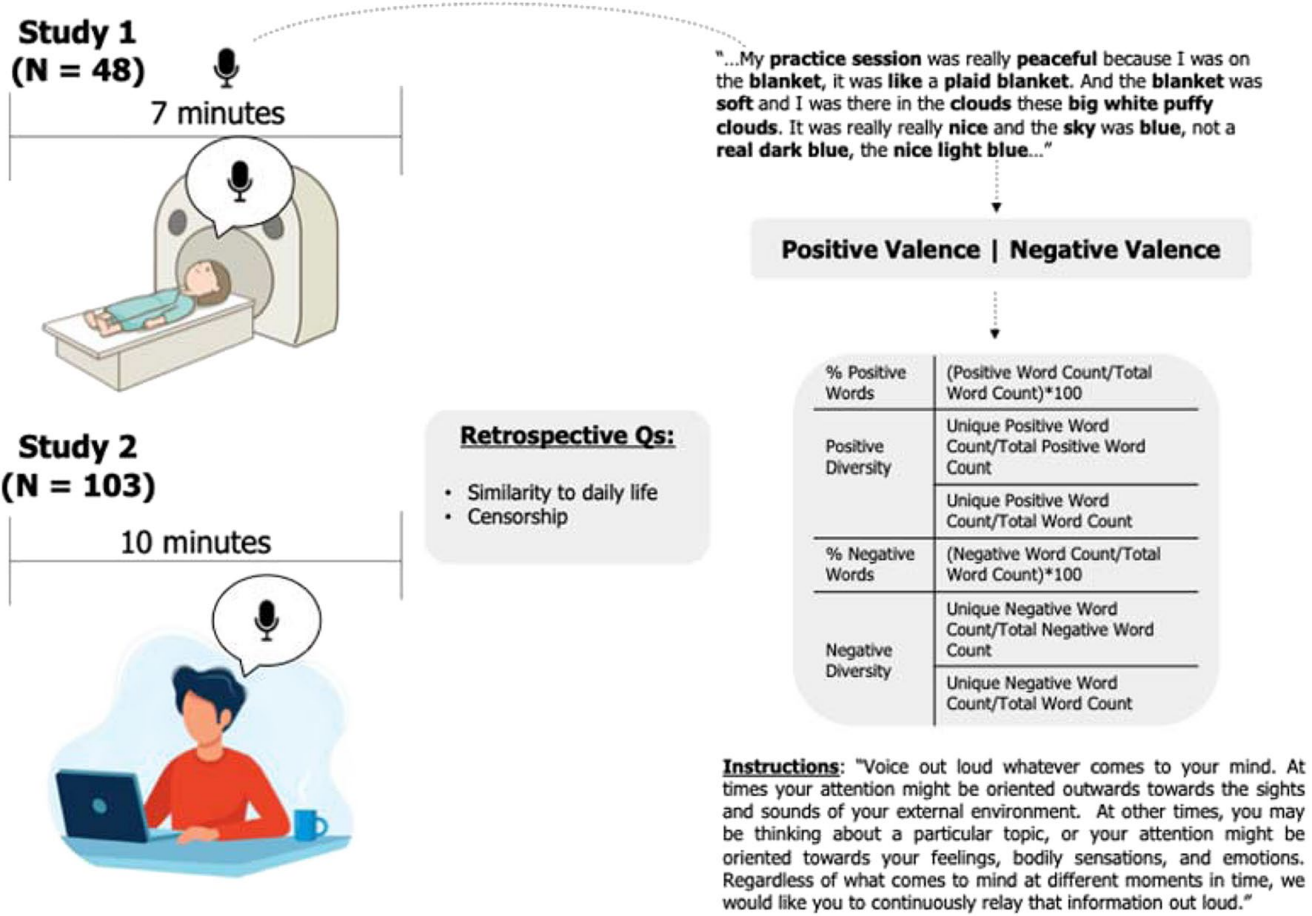
Think-Aloud paradigm. Participants were audio recorded while voicing aloud their resting-state thoughts for 7 min in study 1 (in the MRI scanner) and 10 min for study 2 (at home via Zoom Health). Audio recordings were then transcribed and referenced to an English database of 14,000 English words (Warriner et al., 2013). Words included in the database are bolded in the transcript excerpt. Words were then classified into positively and negatively valenced words using normed valence metrics provided by the Warriner et al. database, and metrics including % positive/negative words and positive/negative diversity metrics were computed

To examine the affective language used to describe resting state thought, we employed the think-aloud paradigm. In study 1, participants practiced speaking their thoughts out loud before the task and were then audio recorded as they spoke aloud the contents of their conscious experience while in an MRI scanner for 7 min (Fig. 1; neuroimaging data not analyzed here).

Previous research has used similar think-aloud paradigms (Li et al., 2022; Raffaelli et al., 2021; Samson et al., 2015; van Calster et al., 2017), but unlike these studies, participants in study 1 were laying down and encouraged to continuously voice aloud whatever came to their mind in a noisy MRI environment, including internal thoughts or images, any perceptions of external stimuli, or any bodily sensations or feelings such as aches, pain, or hunger (similar to a written version of the task in Pennebaker & King, 1999). To encourage participants not to censor themselves, participants were informed that we would turn the volume of the microphone down so that the experimenters could not hear their audio, and a different experimenter than the one collecting their data would transcribe it at a later time (a similar approach was adopted for the virtual environment of study 2, see Supplemental Materials for complete instructions). Following the think-aloud paradigm, participants answered several self-report questions using a continuous sliding scale. Four questions of relevance to the present manuscript were: “How similar were your thoughts to those you experience in your day-to-day life?” “To what extent did you censor yourself during the task?” “How positive were your thoughts?” and “How negative were your thoughts?” The sliding scale’s default position was the midpoint between “0-Not at all” and “1-Extremely.” The final location of the scale was coded as a two-decimal number between .00 and 1.00. Due to technical difficulties with the recording software, some self-report data are incomplete, so we report our sample sizes within each analysis.

Participants also answered the 18-item Ryff Psychological Well-being Scale (18-item version) (Ryff & Keyes, 1995). The scale includes three items for each of six aspects of well-being: self-acceptance, autonomy, environmental mastery, purpose in life, positive relations with others, and personal growth. A total score was computed from the sum of the ratings, coded such that a higher score reported in the manuscript indicates greater well-being. Research has demonstrated the Ryff Well-being Scale is reliable and suitable for assessing well-being (Bayani & Koocheky, 2008).

#### Quantifying the Affective Tone of Language from the Think-Aloud Paradigm

Audio data from the think-aloud paradigm were manually transcribed and marked for non-fluencies and filler words (details in Supplemental Materials), and total words spoken by each participant were computed. Next, we used an independent, previously published database of 13,915 normed English words (Warriner et al., 2013) to construct positive and negative valence word dictionaries, which were then referenced to participants’ unprompted thoughts. The words in the Warriner study were rated by 1,827 young and old participants (age range 16–87 years) for both valence and arousal on a scale that ranged from 1 (unpleasant) to 9 (pleasant) (Warriner et al., 2013). We binned words in the Warriner database into three equally divided categories according to valence: negative (valence rating 1–4.48, 4,103 words, mean valence/arousal = 3.48, 4.58), positive (valence rating 5.52–9, 5,373 words, mean valence/arousal = 6.29, 4.15), and neutral (valence rating 4.49–5.51, 8,542 words, mean valence/arousal = 5.05, 3.93). These bins were created by calculating an even split of 3.48 points above and below the neutral category, represented by 1 center point. The neutral category was not included in subsequent analyses since we were primarily interested in positive and negative naturalistic vocabularies.

We decided to use the Warriner affective database as opposed to other databases, such as those in Linguistic Inquiry Word Count (LIWC) (Tausczik & Pennebaker, 2010) and Vocabulate (Vine et al., 2020), for two main reasons. First, we were concerned that the restricted number of words in other affective dictionaries may not fully capture the diverse repertoire of affectively tinged words used in natural language—a primary goal of our study. For example, the LIWC emotion dictionaries include 406 positive and 499 negative affectively tinged words, and Vocabulate only includes a subset of 53 positive and 92 negative words used to describe emotional feeling states (e.g., happy). In contrast, applying the tercile thresholds described above to the Warriner database yields 5,373 positively tinged English words and 4,103 negatively tinged words. Second, in contrast to LIWC and Vocabulate, the Warriner dictionaries also offer normative quantitative ratings of word valence, allowing us to additionally examine the mean valence of words used, beyond overall percentages.

To quantify the emotional tone of the unprompted thought narratives, we used custom R code to assess both the frequency and diversity of affective word use by referencing the narratives to the Warriner et al., 2013 dictionaries. We calculated overall percentage of positive and negative words, as well as positive and negative linguistic diversity. As our measure of diversity, we determined the proportion of total positive and total negative words that were *unique.* For example, two separate instances of the word “win” in each narrative were only counted once for the metric of positive diversity. Thus, this ratio (unique positive words/total positive words and unique negative words/total negative words) controls for the overall positivity or negativity of each narrative, which we deem important considering that we independently assessed the overall proportion of positivity and negativity. In total, four primary metrics of affective linguistic tone were computed (see Fig. 1): *% positive words* (positive word count/total word count), *% negative words* (negative word count/total word count), *positive diversity* (unique positive word count/total positive word count), and *negative diversity* (unique negative word count/total negative word count). Beyond these primary metrics, two exploratory secondary metrics of diversity were also computed that did not control for differences across participants in the number of positive or negative words: *positive diversity—secondary* (unique positive word count/total word count) and *negative diversity—secondary* (unique negative word count/total word count). These metrics used the identical formula employed by the Vocabulate software (Vine et al., 2020) and differed in their denominator from our main metrics; they were computed simply for readers’ reference to this previous study. Results of these secondary diversity metrics are presented in the Supplemental Materials (Table 4).

#### Statistical Analyses

Throughout the manuscript, we performed two-tailed Student’s *t* tests on variables that were normally distributed or Mann–Whitney *U* tests on variables that were not normally distributed. We report both mean and median, and statistical power is reported as Rank-Biserial Correlation (RBC) or Cohen’s *d*. Although outlier removal is not necessary with nonparametric statistics (Corder & Foreman, 2014), we performed outlier removal with SPSS (a step of 1.5 × Interquartile range) in the first processing steps of the analyses.

## Study 1 Results

### Well-being

**Fig. 2:**
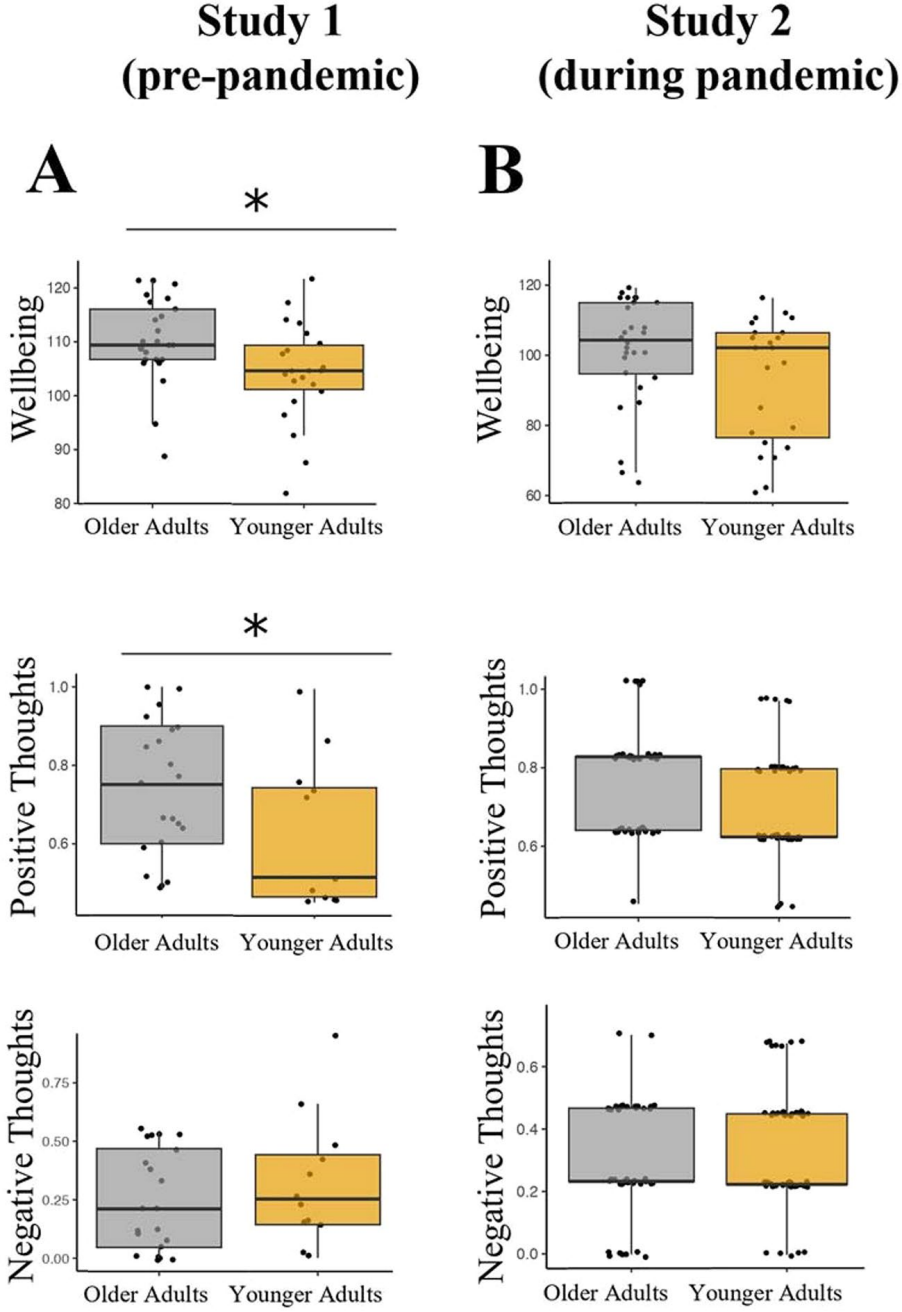
Age differences by study in well-being and self-reported positive and negative thoughts. In study 1 (**A**), older adults reported higher well-being (top) and more positive thoughts (middle) during the think-aloud paradigm compared to young adults. No age differences were noted in self-reported negative thoughts (bottom). In study 2 (**B**), there were no age differences in well-being (top), in self-reported positive (middle) or negative thoughts (bottom). ^a^Box plots depicts median, not mean. *p < .05

We first aimed to establish whether older adults self-reported higher well-being as expected based on prior research (Blanchflower & Oswald, 2008; Kim et al., 2021; Smith & Hollinger-Smith, 2015). We conducted an independent samples *t* test on total scores from the Ryff Well-being Questionnaire across age groups. Older adults (*Mdn* = 102, *M* = 103) scored higher than younger adults on the Ryff Psychological Well-being Questionnaire (*Mdn* = 97, *M* = 96.4), *U* = 178, *p* = .039, *RBC* = .353 (Fig. 2A, top), indicating higher well-being in older adults.

### Think-Aloud Paradigm

#### Self-Reported Thoughts

To understand how participants interpreted the think-aloud paradigm, we assessed their perceived positivity or negativity of their spoken thoughts, whether those thoughts were similar to those in their everyday life, and to what extent they self-censored their thoughts﻿. Older adults (*Mdn* = .751, *M* = .740, *N* = 21) retrospectively reported having significantly more positive thoughts overall during the think-aloud paradigm than young adults (*Mdn* = .494, *M* = .572, *N* = 12), *U* = 66, *p* = .026, *RBC* = .476 (Fig. 2A, middle). There were no age differences in the amount of self-reported negative thoughts between older adults (*Mdn* = .200, *M* = .233, *N* = 21) and young adults (*Mdn* = .240, *M* = .307, *N* = 12), *U* = 105, *p* = .442, *RBC* = .167 (Fig. 2A, bottom).

Resting-state thoughts were rated moderately similar to everyday life, which did not differ between older (*Mdn* = .523, *M* = .560, *N* = 10) and young adults (*Mdn* = .656, *M* = .572, *N* = 7), *U* = 34, *p* = .962, *RBC* = .028. Self-censoring during the think-aloud paradigm was low for both age groups and did not significantly differ across groups (old: *Mdn* = .100, *M* = .140, *N* = 21; young: *Mdn* = .082, *M* = .190, *N* = 16; *U* = 166.5, *p* = .975, *RBC* = .009). Finally, the total number of words older adults spoke during the think-aloud paradigm (*Mdn* = 873, *M* = 818) was statistically similar to those spoken by young adults (*Mdn* = 788, *M* = 829), *U* = 277, *p* = .860, *RBC* = .032. Collectively, these findings indicate that resting-state thought variables have similar degrees of ecological validity between age groups. Having established the details of the resting-state thought context, we next investigated whether age differences existed in the affective tone of participants’ naturalistic vocabularies used to describe their thoughts.

#### Affective Linguistic Diversity

**Fig. 3:**
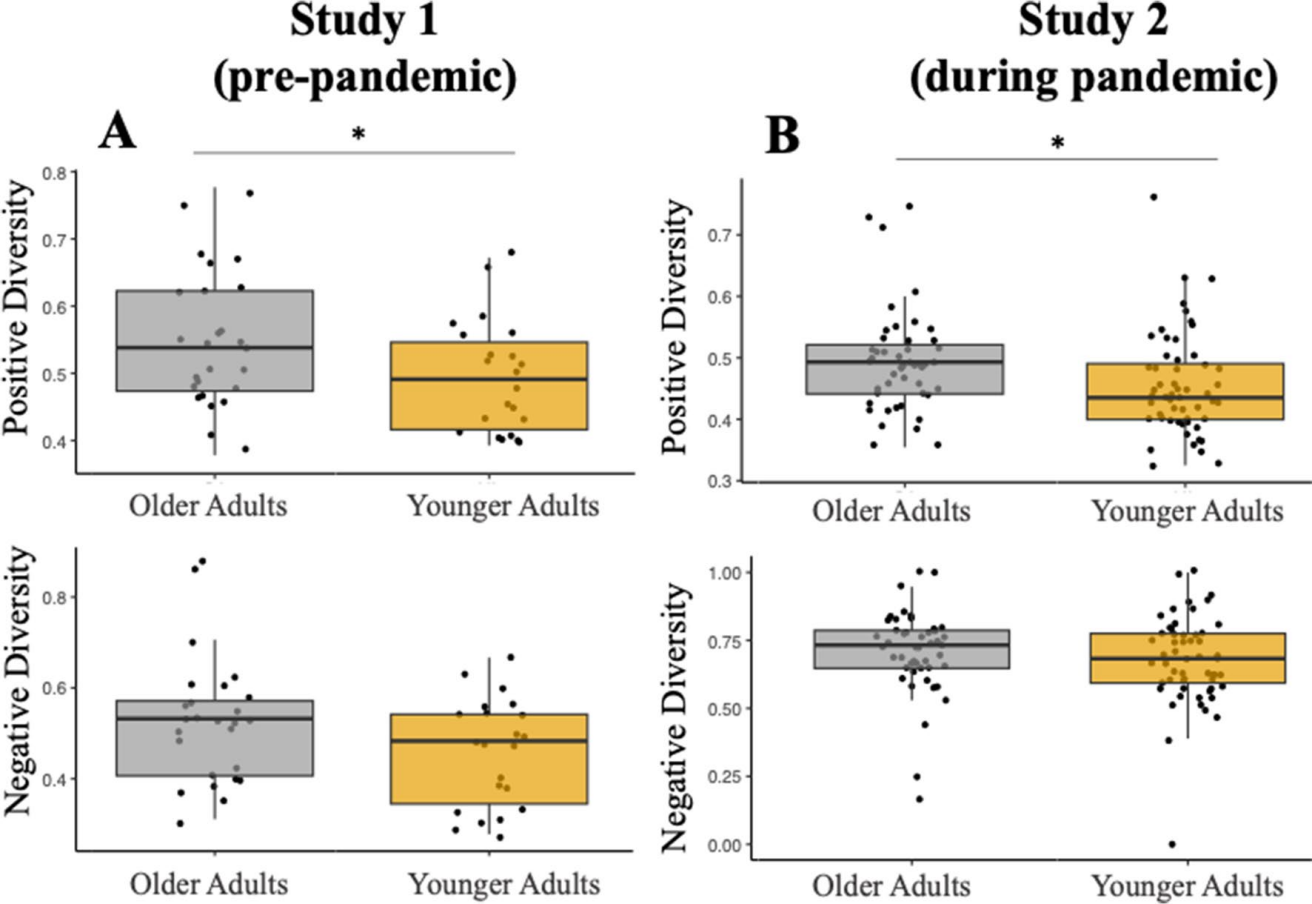
Age differences by study in positive and negative diversity. In study 1 (**A**), older adults exhibited higher positive diversity compared to young adults (top), as measured by larger proportion of unique positive words/total positive words spoken, but no differences were noted in the proportion of unique negative words/total negative words spoken (negative diversity; bottom). In study 2 (**B**), we replicate these findings. Note that box plots depict median, not mean. *p < .05

Older adults exhibited more positive diversity of their vocabularies compared to young adults (older adults: *M* = .550, younger adults: *M* = .493, *t*(46) = 2.13, *p* = .039, *d* = .616, 95% CI [.018, 1.20]). However, older adults did not significantly differ from younger adults in their negative diversity, the ratio of unique negative words to total negative words (older adults: *Mdn* = .532, *M* = .527, younger adults: *Mdn* = .483, *M* = .458,* U* = 209, *p* = .111, *RBC* = .271, Fig. 3B).

We further explored whether these diversity results were paralleled by the percentage of positive or negative words used or the positive or negative valence/arousal of words spoken by each participant.

#### Frequency of Affective Word Use, Mean Valence, and Mean Arousal

In contrast to the self-reported positivity and the diversity of affective vocabularies, no significant difference in groups emerged in other measures of affective language. More specifically, in contrast to our hypothesis, there were no significant age differences in the percent of positive words generated between older (*Mdn* = .216, *M* = .222) and young adults (*Mdn* = .197, *M* = .215; *U* = 232, *p* = .271, *RBC* = .189), nor in the percent of negative words generated between older (*Mdn* = .038, *M* = .039) and young adults (*Mdn* = .038, *M* = .041; *U* = 257, *p* = .559, *RBC* = 101). Furthermore, the mean valence of words generated for older adults (*Mdn* = 6.14, *M* = 5.80) did not differ from young adults (*Mdn* = 6.11, *M* = 5.79; *U* = 402,161, *p* = .735, *RBC* = .009). Similarly, the mean arousal of words generated did not differ between older (*Mdn* = 4.10, *M* = 5.80) and young adults (*Mdn* = 4.17, *M* = 4.25; *U* = 388,852, *p* = .124, *RBC* = .042). Thus, despite comparable use of valence/arousal and positive/negative words, older adults exhibited more positive diversity of their naturalistic vocabularies.

### Study 1 Summary

First, we show that older adults have higher self-reported well-being compared to young adults, which replicates a breadth of previous research on cognitively unimpaired Americans (Baltes & Baltes, 1990; Bowling, 2011; Hazzard et al., 2003; Stone et al., 2010). Second, we demonstrate that during resting-state thought, older adults were more positively diverse in their affectively tinged vocabularies compared to young adults; that is, they spoke a larger proportion of unique positive words out of both total and only positive words compared to their younger counterparts. Third, we found that older adults self-reported having more positive thoughts during the think-aloud paradigm compared to young adults. Taken together, these results provide evidence that the positivity effect may persist during periods of unconstrained thought. The results of study 1 also suggest that participants did not appear to censor themselves or to experience the think-aloud paradigm as overtly artificial, which contributes to its ecological validity.

We next tested the replicability of these results in a larger sample. Due to the unpreceded nature of the COVID-19 pandemic, the second study had two important distinctions. First, the think-aloud paradigm was conducted virtually due to health concerns. Second, based on the assumption that older adults may have a larger vocabulary, we added a vocabulary knowledge test and assessed the relationship between vocabulary and diversity metrics.

## Study 2

### Study 2 Method

A total of 114 younger and older participants were recruited from the University of Arizona and greater Tucson communities. Electronic informed consent was obtained from all participants, and all procedures were performed in accordance with regulations and approved by the University of Arizona’s Institutional Review Board. In line with our previous research (Wank et al., 2021), older adults were comprehensively screened for abnormal age-related cognitive decline using a profile actuarial approach (Edmonds et al., 2014). Domains tested were memory (California Verbal Learning Test Second Edition, Long Delay Free Recall; Rey Complex Figure Test), language (Boston Naming Test Total Score; Animal Fluency Total Correct), and attention/executive functioning (21 participants completed Trail Making Test A&B in person before the pandemic began; the remaining 35 participants completed the Digit Span Forward Total Score and Digit Span Backward Total Score from the Wechsler Adult Intelligence Scale IV, which was better suited for the virtual format). Consistent with prior research (Edmonds et al., 2014; Wank et al., 2021), two scores of more than 1 standard deviation below the age and education corrected mean in one domain or three scores across the three domains were used to screen out potential mild cognitive impairment. Based on these criteria, two older participants were excluded. Nine participants’ TA audio files were inaudible. Thus, the total analyzed sample included 55 young adults (74.5% female; age range: 18–34; mean age 22.6 years old) and 48 older adults (72.9% female; age range: 61–83 years old; mean age 69.6 years). We also collected participant’s perceived socioeconomic status (SES) using the MacArthur Scale of Subjective Social Status (Table 1, study 1 methods, details in Supplemental Material), as well as participants’ employment information (YA: student 53%; unemployed 5%; part-time11%, full-time 27%; OA: unemployed 2%, part-time 10%, full-time 10%, retired 76%). No age group differences were observed in subjective SES. See Table 1 for detailed sociodemographic information.

#### Virtual Think-Aloud Paradigm

Because Study 2 occurred during the height of the COVID-19 pandemic, the think-aloud paradigm was conducted virtually using Zoom Health. The major difference in study 2 in the virtual paradigm was that participants were instructed to speak aloud their thoughts from the comfort of their own home (as opposed to in the MRI scanner) and for 10 min (instead of 7 min; Fig. 1). Similar to study 1, participants also completed a practice run to familiarize themselves with the process of thinking aloud and were encouraged not to censor themselves while thinking or speaking (see Supplemental Materials for details on self-reported censorship metrics and Think-Aloud adaptation to virtual environment). The audio files were transcribed and analyzed in the same way as in study 1.

#### Assessing Psychological Well-being

To assess psychological well-being, the 18-item Ryff questionnaire, also included in study 1, was administered to study 2 participants. However, the full questionnaire was added half-way through the study and was thus only available for a subset of 49 out of 103 participants.

#### Assessing Vocabulary Knowledge

In study 2, we assessed vocabulary knowledge with the vocabulary subtest from the Wechsler Adult Intelligence Scale IV (WAIS, Wechsler, 2008). This allowed us to examine how vocabulary contributed to metrics of linguistic affective diversity.

#### Quantifying Affective Language and Statistical Analyses

A key aim of study 2 was to determine the replicability of results from study 1; therefore, we implemented identical data preparation and analysis procedures.

#### Study 2 vs. Study 1 Comparisons

We compared both the experience of the think-aloud paradigm and affective language metrics across studies by performing a series of Mann–Whitney *U* tests given the non-normal distributions of the data. The scales of thought censorship, everyday thoughts similarity, and self-reported positive and negative thoughts were different from study 1 (0–1 scale) to study 2 (1–5 scale); therefore, we converted study 2 scores to the same scale as study 1.

### Study 2 Results

Overall, we largely replicate the results of study 1 but state the few differences that emerged within each relevant section.

#### Well-being

Only a subset of 49 participants in study 2 received the 18-item Ryff well-being questionnaire. Similar to study 1, older adults (*Mdn* = 113 M = 110) scored numerically higher on the Ryff psychological well-being measure compared to young adults (*Mdn* = 111, *M* = 105), but unlike study 1, this difference was not significant (*U* = 237, *p* = .109, *RBC* = .264 (Fig. 2B, top). Overall well-being decreased significantly in study 2 (*Mdn* = 92 M = 89.8) compared to study 1 (*Mdn* = 101* M* = 100), *U* = 573, *p* < .001, *RBC* = .502).

#### Self-reported Thoughts

Although older adults’ self-reported positive thoughts (*Mdn* = .750, *M* = .660) were numerically higher compared to young adults (*Mdn* = .500, *M* = .609), this difference was not significant in study 2 as it was in study 1, *U* = 1,100, *p* = .158, *RBC* = .149 (Fig. 2B, middle). There was also no significant difference in older adults’ negative thoughts (*Mdn* = .500, *M* = .609) compared to young adults (*Mdn* = .500, *M* = .319), *U* = 1,149, *p* = .299, *RBC* = .111 (Fig. 2B, bottom). Overall, participants self-reported a statistically similar degree of positive thoughts across both studies, but self-reported more negative thoughts during study 2 compared to study 1 (Table 2).

Replicating study 1, there were no differences in participants’ self-reported similarity of their resting-state thoughts to those experienced in everyday life between older (*Mdn* = .750, *M* = .745) and young adults (*Mdn* = .750, *M* = .714), *U* = 1,180, *p* = .331, *RBC* = .106. However, unlike study 1, young adults (*Mdn* = .250, *M* = .345) reported censoring themselves to a greater degree than older adults (*Mdn* = .250, *M* = .161) during the think-aloud paradigm, *U* = 738, *p* < .001, *RBC* = .441. Overall, participants reported more similarity to everyday thoughts in study 2 compared to study 1 (perhaps because of their home environment), but interestingly, more self-censoring as well (Table 2). Importantly, however, self-censorship was not related to either study’s main outcome variables (Supplemental Material Tables 1 and 2), many of which differ across age groups.

**Table 2:** Experience of task characteristics in study 1 and 2

	Study 1			Study 2			Study 1-study 2 differences	
Self-reported variable	*Mean*	*SD*	*Median*	*Mean*	*SD*	*Median*	*Significance test*	*p value*
Everyday thought similarity	.565	.258	.626	.728	.235	.750	*U* = 532	.008**
Self-censoring	.161	.209	.083	.260	.224	.250	*U* = 1,420	.017*
Self-reported positive thoughts	.679	.219	.663	.632	.185	.500	*U* = 1,489	.302
Self-reported negative thoughts	.260	.227	.209	.346	.250	.202	*U* = 1,277	.031*

Statistical differences between study 1 and 2 were evaluated with a Mann–Whitney *U* test for non-normally distributed variables

*p < .05, **p < 0.01

#### Affective Linguistic Diversity

Replicating study 1, older adults (*Mdn* = .493, *M* = .494) exhibited more positive diversity in their vocabularies to describe their resting state thoughts compared to young adults (*Mdn* = .435, *M* = .455) (unique positive words/total positive words, *U* = 896, *p* = .005, *RBC* = .322 (Fig. 3B, *top*). Like study 1, there were no significant differences in negative diversity between older and younger adults (older adults *Mdn* = .759, *M* = .931; young adults: *Mdn* = .634, *M* = .821), *U* = 1,175, *p* = .338, *RBC* = .110 (Fig. 3B, *bottom*). When directly comparing the two studies, participants in study 1 were *more* positively diverse and *less* negatively diverse than participants in study 2 (Table 3).

As in study 1, we explored whether these replicated results were driven by the percentage of positive or negative words used, or the positive or negative valence/arousal of words spoken by each participant.

#### Frequency of Affective Word Use, Mean Valence, and Mean Arousal

Replicating study 1, there were no differences in the percent of positive words used between older (*Mdn* = .189, *M* = .230) and young adults (*Mdn* = .188, *M* = .223), *U* = 1,296, *p* = .877, *RBC* = .018, nor in the percent of negative words used between older (*Mdn* = .189, *M* = .230) and young adults (*Mdn* = .188, *M* = .223), *t*(101) =  − .247, *p* = .805, *d* =  − .048, 95% CI [− .435 .339]. Similarly, we found no age differences in the mean valence of words spoken between older (*Mdn* = 6.18, *M* = 6.12) and young adults (*Mdn* = 6.16, *M* = 6.17), *t*(101) =  − 1.52, *p* = .136, *d* =  − .296. No differences in arousal of words spoken between older (*Mdn* = 3.98, *M* = 3.98) and young adults (*Mdn* = 4.04, *M* = 4.01), *U* = 1,042, *p* = .064, *RBC* = .212, emerged either.

Beyond study 2’s findings, valence and arousal measures were statistically similar between studies. Additionally, participants in study 2 spoke a significantly greater percentage of negative words than study 1 but had a significantly less negative diversity compared to study 1. No study differences emerged in the percentage of positive words spoken (Table 3).

**Table 3:** Affective tone metrics in study 1 and 2

	Study 1			Study 2			Study 1-study 2 differences	
	*Mean*	*SD*	*Median*	*Mean*	*SD*	*Median*	*Significance Test*	*p value*
Mean Valence	6.210	.186	6.210	6.150	.162	6.160	*U* = 2,030	.078
Mean Arousal	4.030	.127	4.020	4.000	3.990	.094	*U* = 2,108	.146
% Positive Words	.219	.061	.203	.226	.136	.188	*U* = 2,058	.098
% Negative Words	.040	.014	.038	.022	.008	.021	*U* = 561	< .001**
Positive Diversity (Unique Pos/Total Pos)	.523	.096	.509	.472	.085	.459	*U* = 1,677	.001**
Negative Diversity (Unique Neg/Total Neg)	.495	.132	.500	.872	.609	.666	*U* = 1,474	< .001**

Statistical differences between study 1 and 2 were evaluated with a Mann–Whitney *U* test for non-normally distributed variables

*p < .05, **p < .01

#### Post Hoc Analyses of Content

To gain insight into why participants in study 2 self-reported more negative thoughts and were more negatively but less positively diverse in their resting state thoughts, we assessed the content of participants’ think-aloud transcripts by identifying the most frequently used negative and positive words for each study. Interestingly, high-frequency negatively valenced words used in study 2 tended to revolve around pandemic-related concerns. These included words such as “boring,” “anxious,” “nervous,” “worried,” “hospital,” and “sick,” which were used most often for both younger and older adults in study 2. Words used at high frequency in study 1 for both older and younger adults seemed to revolve around the uncomfortable scanner environment (e.g., “noise,” “hard,” “cold,” “jackhammer”). In study 1, 95% of participants mentioned at least one word related to the scanner environment and 100% of the participants in study 2 used words related to a distressed mindset. Our observation that thought content during the pandemic often related to this important societal concern may have reflected the lower well-being in this sample compared to study 1 (as reported in the “Study 2 Results” above).

In contrast, the nature of the positive words used across studies and age groups was similar, and many words conceptually related to “gains” (e.g., “have”, “get,” “want”) and socially related themes (e.g., “kind”, “people”).

#### Vocabulary Knowledge

Older adults (*Mdn* = 49, *M* = 49.10) had significantly higher vocabulary knowledge compared to young adults (*Mdn* = 47, *M* = 45.7), *U* = 889, *p* = .014, *RBC* = .28. Despite these group differences, vocabulary knowledge did not significantly relate to positive nor negative diversity, either when controlling for age group (*positive diversity:* Spearman’s rho (98) =  − .19, *p* = .059; *negative diversity:* Spearman’s rho (98) =  − .16, *p* = .12), or without controlling for age group (*positive diversity:* Spearman’s rho (98) =  − .10, *p* = .32; *negative diversity:* Spearman’s rho (98) =  − .13, *p* = .21) (Supplemental Material Tables 3 and 4). Additionally, older adults remained significantly higher on positive diversity when adjusting for vocabulary knowledge by including it as a covariate in an analysis of covariance (ANCOVA) (*t*(97) = 3.08, *p* = .003, Cohen’s *d* = .64; *age group: F*(1,97) = 9.49, *p* = .003, *η*^*2*^ = .086; *vocabulary knowledge: F*(1,97) = 3.54, *p* = .063, *η*^*2*^ = .032). Finally, despite age-related differences in vocabulary, we found no significant difference in the number of words older adults spoke during the think-aloud paradigm (*Mdn* = 1,371, *M* = 1,295) compared to those spoken by young adults (*Mdn* = 1,212, *M* = 1,197), *U* = 1,075, *p* = .106, *RBC* = .186, replicating study 1 findings. Overall, these findings suggest that age group differences in positive diversity cannot be accounted for either by overall words spoken or by age-related increases in vocabulary knowledge.

### Study 2 Summary

In study 2, we largely replicate study 1 results in that older adults used a more varied repertoire of positive words when describing their resting state thoughts. Additionally, despite older adults having higher general vocabulary knowledge compared to young adults on an established neuropsychological test, general vocabulary was not related to any affective language metrics.

## Relationships with Well-being Across Aggregated Study Samples

### Aggregated Study Methods

Finally, to examine whether positive diversity related to well-being, we combined data from both studies to boost power for these individual difference analyses and controlled for study cohort. In total, 96 participants across studies completed the same 18-item Ryff Questionnaire to measure well-being, including 44 young adults (57% female, age range: 19–35, mean age 25.8 years old) and 52 older adults (67.2% female, age range: 58–82 years old, mean age 68.3 years old). All variables in subsequent analyses were *z*-scored.

### Aggregated Study Results

In the aggregated sample controlling for study, older adults (*M* = .196, *Mdn* = .194) exhibited significantly higher well-being scores than younger adults (*M* =  − .231, *Mdn* =  − .227), *U* = 821, *p* = .018, *RBC* = .265, as well as significantly higher positive diversity (OA: *M* = .299, *Mdn* = .222, YA: *M* =  − .353, *Mdn* =  − .473, *U* = 701, *p* = .001, *RBC* = .337). In contrast, neither negative diversity (negative diversity: OA: *M* = .100, *Mdn* = .095, YA: *M* =  − .118, *Mdn* =  − .045, *U* = 950, *p* = .155, *RBC* = .163), percent positive words (OA: *M* = .096, *Mdn* =  − .169, YA: *M* =  − .114, *Mdn* =  − .215, *U* = 1,126, *p* = .898, *RBC* = .052), nor percent negative words (OA: *M* =  − .053, *Mdn* =  − .199, YA: *M* = .063, *Mdn* =  − .077, *U* = 1,023, *p* = .374 *RBC* = .126) were significantly different across age groups.

**Fig. 4:**
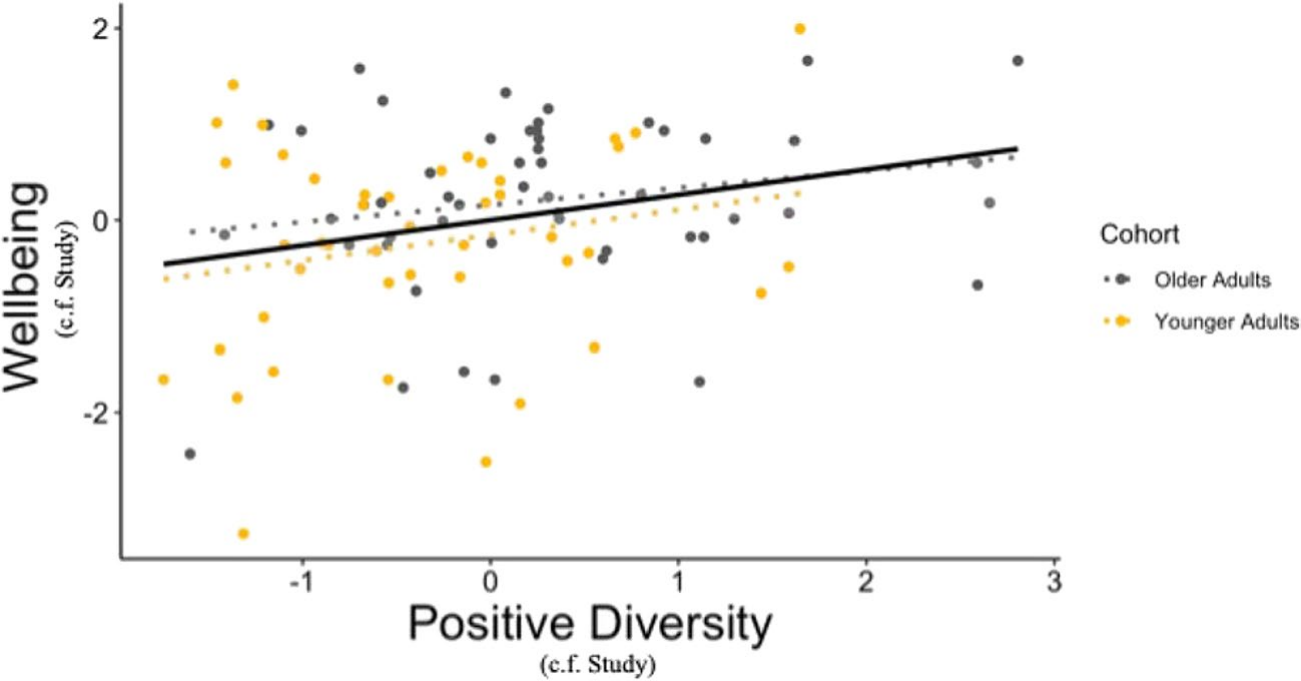
Relationship between positive diversity and well-being: The relationship between positive diversity and well-being in participants aggregated across studies (residuals after controlling for study cohort) was significantly correlated (Spearman’s rho (94) = .23, *p* = .023). Note that a multiple regression analysis described in the main text revealed that this relationship did not significantly interact with age. However, for visual purposes only, we also plotted the relationship in each group separately in dotted lines. C.f.: controlled for study. Interval represents standard error

We next assessed whether well-being was associated with positive diversity when controlling for study environment. We found that well-being was positively correlated with positive diversity (unique positive words/total positive words), Spearman’s rho (94) = .23, *p* = .023 (Fig. 4).

Finally, to additionally assess the role of age and other metrics of affective language, we performed a stepwise regression with well-being as the dependent variable.

As detailed in Table 4, we included age and study cohort as predictors of well-being in an initial step, revealing an overall significant model (*p* < .001) and significant independent effects of both predictors (age *p* = .012, study *p* < .001). To examine whether positive diversity added independent variance, we added positive diversity to the model in a second step, resulting in a significantly improved fit (∆*R*^2^ = .035, *p* = .044), and a significant independent positive effect of positive diversity (*p* = .044). Interestingly, with the addition of positive diversity, the effect of age became weaker and lost its significance (*p* = .20), suggesting that in this study, the positive diversity metric largely accounts for the relationship between age and well-being. To examine whether the relationship between positive diversity and well-being varied with age, we added an age × positive diversity interaction term in a third step. The interaction term was not significant (*p* = .62), and the model did not improve the fit (∆*R*^2^ = .0021, *p* = .62), suggesting a similarly positive relationship between diversity and well-being across age. Finally, to examine the influence of positive and negative frequency, we added positive words/total words and negative words/total words in a final step. Again, the model fit did not significantly improve (∆*R*^2^ = .011, *p* = .82) and neither metric of positive nor negative affective language significantly independently predicted well-being (positive % *p* = .51, negative % *p* = .68). Importantly, positive diversity remained a significant independent predictor (*p* = .036) in the final model.

**Table 4:** Stepwise regression results using well-being as predictor

		*b*		*beta*			
Predictor	*b*	95% CI	*beta*	95% CI	*r*	Fit	Difference
		[LL, UL]		[LL, UL]			
(Intercept)	0	[− .18,.18]					
Age	.194*	[.00, .38]	.19	[.00, .38]			
Study	− .398**	[− .58, − .21]	− .40	[− .58, − .21]			
					.441**	*R*^2^= .194**	
(Intercept)	0	[.18, .18]					
Age	.1250	[− .06, .32]	.13	[− .08, − .31]			
Study	− .35**	[− .54, − .17]	− .35	[− .53, − .16]			
Positive diversity	.204*	[.01, .40]	.200	[.01, .40]			
					.479**	* R*^2^= .229**	ΔR^2^= .035*
(Intercept)	.016	[− .17, .21]					
Age	.122	[− .07, .32]	.12	[− .07, − .32]			
Study	− .35**	[− .53, − .16]	− .35	[− .54, − .16]			
Positive diversity	.21*	[.01, .42]	.21	[.01, .42]			
Age*Pos Div	− .05	[− .24, .14]	− .05	[− .24, .14]			
					.481**	* R*^2^= .232**	ΔR^2^=.002
(Intercept)	.005	[− .19, .20]					
Age	.120	[− .08, .32]	.12	[− .08, .32]			
Study	− .310*	[− .55, − .06]	− .31	[− .55, − .06]			
Positive diversity	.220*	[.01, .43]	.22	[.02, .43]			
Age*Pos Div	.100	[− .22, .19]	− .01	[− .22, .19]			
% positive words	.070	[− .14, .28]	.07	[− .14, .28]			
% negative words	.050	[− .20, .31]	.05	[− .20, .31]			
					.489**	* R*^2^= .239**	ΔR^2^=.011

A significant *b*-weight indicates the beta-weight and semi partial correlation are also significant. *b* represents unstandardized regression weights; beta indicates the standardized regression weights; *r* represents the zero-order correlation. LL and UL indicate the lower and upper limits of a confidence interval, respectively.

*p < .05; **p < .01

Vocabulary knowledge as measured with the WAIS was only available in study 2 so we did not include vocabulary in the stepwise regression. In the smaller set of participants with both WAIS vocabulary scores and the Ryff well-being measure available (*N* = 48), the relationship between the two variables was positive but not significant (Spearman’s rho (46) = .25, *p* = .087).

### Aggregated Study Discussion

Overall, these results point to an important relationship between positive diversity and well-being, independent of age and other measures of affective language. Our finding that both positive diversity and well-being are greater in older adults, combined with a non-significant interaction with age and an effect of age that appears to be accounted for by positive diversity in the stepwise regression, raises the possibility that positive diversity may represent a cognitive affective mechanism or manifestation of underlying elevated well-being in older adults.

## General Discussion

The present manuscript examined age differences in affective language during idle periods, across two independent studies, before and during the COVID-19 pandemic. In both studies, our findings showed that cognitively normal older adults demonstrated larger positive emotional vocabularities when voicing aloud their unprompted thoughts. Furthermore, across participants, this metric predicted higher well-being independent of other metrics of affective language and did so in a manner that was not moderated by age. Considering that older adults also scored higher on a questionnaire of psychological well-being, our findings raise the speculative possibility that positive diversity may contribute mechanistically to elevated well-being in older adults and may point to an important target of therapeutic intervention across the lifespan.

We found that across our two samples, older adults were more positively (but not more negatively) varied in their resting-state language compared to younger adults, only partially supporting our hypothesis that older adults would exhibit higher positive tone in addition to higher positive diversity. Higher emotional diversity may have adaptive value for mental health by providing psychological flexibility to guide use of emotion regulation strategies in affective contexts (Quoidbach et al., 2014; Barrett et al., 2001; Kashdan & Rottenberg, 2010). Indeed, recent research demonstrates that the diversity of words used to represent positive feeling states is linked to enhanced well-being (Vine et al., 2020), a finding we extend here by showing well-being relationships with the diversity of a much larger repertoire of positive words in unprompted thoughts. Conversely, prior work shows that the impaired ability to disengage from negative words is positively related to rumination in daily life and is negatively related to well-being (Holas et al., 2018; Kaiser et al., 2015; see also Raffaelli et al., 2021). Our findings of enhanced positive linguistic diversity in older adults may be due to the possibility that older adults gain enhanced practice wielding emotional knowledge over time that selecting diverse emotional words to describe their positive thoughts becomes habitual. Indeed, the tendency to rely on acquired knowledge has been robustly paralleled by cognitive decline in other areas (Spreng & Turner, 2019).

Our results did not show age differences in negative affective language. Tangential research exploring the taxonomies of indigenous hunter-gatherers suggests that attention to particular stimuli may contribute to the development of increasingly diverse vocabularies (Zgusta et al., 2006). It may be that older adults’ habitually positive thoughts, as documented by the positivity effect (Mather & Carstensen, 2005), may be partly responsible for a higher number of positive diverse words they naturally speak. Interestingly, we also found that more positive diversity does not significantly relate to a larger overall reserve of vocabulary knowledge, as measured by older adults’ higher vocabulary scores compared to young adults. Therefore, we speculate older adults may be cycling through a larger repertoire of distinct positive thoughts, which may be reflected in the diversity of words expressed during their resting state thought. Taken together with data documenting the positivity effect in attention and memory (Charles et al., 2003; Grühn & Scheibe, 2008; Mather & Carstensen, 2005; Mather et al., 2004; Reed et al., 2014), our results suggest that the positivity bias phenomenon may extend to unique positive words expressed during resting-state thought.

In our aggregated sample analysis, we first show that our quantification of positive diversity (unique positive words/total positive words) is related to overall well-being, irrespective of age and study environment, and as reported in the Supplemental Materials, a previous quantification of positive diversity (unique positive words/total words) does not show this relationship. Second, our metric explains unique variance beyond overall positive affective language. Previous work in linguistics demonstrates that there is moderate stability in how people express themselves linguistically across time, locations, activities, and modes of interaction (Mehl et al., 2001; Mehl & Pennebaker, 2003; Park et al., 2015; Pennebaker & King, 1999), and that between-person differences in naturalistic language use are correlated with differences in outcome measurements such as mental health and longevity (Danner et al., 2001; Guntuku et al., 2017; Hirsh & Peterson, 2009; Rude et al., 2004; Schwartz et al., 2013; Yarkoni, 2010; Ziemer & Korkmaz, 2017). Although some discrepancies between our diversity metrics existed, both showed increases in older adults compared to younger adults, and the metric we chose does not conflate a positive emotional tone due to its calculation. To this end, we assert that the metric presented in this paper is a more refined calculation of emotional diversity in the language of older adults and may be better able to account for subtle differences between the cohorts. Our work extends valuable previous research indicating that positive naturalistic thought may be related to mental well-being (Vine et al., 2020) by quantifying positive diversity in a novel way and suggesting positive diversity may represent a manifestation of the age-related positivity effect, which may be related to improved well-being.

Furthermore, our findings using the automated text analysis software highlight its scalability, benefits of automaticity, and potential relevance in assessing stream-of-consciousness thought in clinical populations. By using a large selection of normed emotional words estimated to be between one half and one quarter of the words known to individuals (Warriner et al., 2013), we increase the reliability of detecting a relationship between personal, naturally occurring vocabularies and their underlying emotional tone. The extensive Warriner et al. dictionaries include 4,967 more positive words and 3,604 more negative words than LIWC (Tausczik & Pennebaker, 2010). They also extend beyond feeling states, which is the focus of Vocabulate software (Vine et al., 2020) and offer normed ratings across the lifespan of word valence, arousal, and dominance. Therefore, this approach may allow for a richer and more accurate quantification of the underlying diversity of unprompted thought.

Prior studies investigating affective language in open-ended scenarios (Greasley et al., 2000; Ottenstein & Lischetzke, 2020; Williams & Uliaszek, 2021) gave credence to the resting state as invaluable terrain for assessing affective thought patterns. Importantly, measuring diversity during unplanned spontaneous speech (versus responses to writing prompts), as we did here, has the advantage of being used in settings such as neuroimaging environments where writing may be difficult (Li et al., 2023). Furthermore, since participants are not unduly influenced by itemized emotional categories, this metric yields fruitful links to not only individual differences in the diversity of resting-state thought, but also a broader understanding of how emotional words may create positive moods. Our findings furthermore lend support to studies showing that older adults experience more positive unprompted thoughts during laboratory tasks and in their daily life (Irish & Piguet, 2013; Irish et al., 2019; Maillet et al., 2018; Turnbull et al., 2021).

Resting-state thought has mainly been characterized indirectly, through retrospective questionnaires (Diaz et al., 2013; Delamillieure et al., 2010), or defined as brain activity in the *absence* of task or external stimulation, instead of being the sole focus of inquiry (Callard & Margulies, 2014). However, despite being the target of many neuroimaging investigations (i.e., resting state connectivity), experiential characteristics associated with resting state contexts are only now starting to be explored (e.g., Hurlburt et al., 2015; Karapanagiotidis et al., 2020; Li et al., 2022; van Calster et al., 2017; Vatansever et al., 2020). Although the age-related positivity effect has been explored during a range of cognitive tasks (Isaacowitz et al., 2006; Kennedy et al., 2004; Löckenhoff & Carstensen, 2008; Mather & Carstensen, 2003; Mikels et al., 2005), our study is one of the first to show that the positivity effect persists during periods of unconstrained thought. As demonstrated here and by others (Pennebaker & King, 1999; Raffaelli et al., 2021; Tausczik & Pennebaker, 2009), the think-aloud paradigm is a short, convenient, and ecologically valid paradigm with convergent and predictive validity that significantly adds to our understanding of internal thought content. We show that the think aloud paradigm can be utilized in myriad environments given that participants’ similarity to everyday thoughts remained high. Additionally, the think aloud paradigm has the advantage of avoiding issues related to retrospective and subjective assessment of thought, which is common in other resting state paradigms.

We append to a multitude of previous research showing that older adults report higher psychological well-being than young adults (Carstensen, 2006; Urry & Gross, 2010), a result that held during a global pandemic (Carstensen et al., 2020). Additionally, older adults self-reported experiencing more positive thoughts than young adults in study 1 (but not in study 2). The global lockdown led to increased incidence of mental illness, suicide, anxiety, depression, loneliness, and social isolation (Cullen et al., 2020; Kontoangelos et al., 2020; Lwin et al., 2020; Pfefferbaum & North, 2020). Although older adults were at the greatest risk of illness from COVID-19 and experienced longer and more restrictive periods of social isolation, previous evidence suggests this population possesses remarkable emotional resilience in the face of pandemic stressors (Carstensen et al., 2020). One study investigating positive valence ratings of 3,600 English words showed that older adults produced higher positive valence word ratings overall than their younger counterparts before and especially during the pandemic (Kyröläinen et al., 2021). In keeping with this research, we suggest our findings, which may reflect evidence of the positivity effect in older adults, persist during periods of heightened stress, and we speculate that positive diversity may be an emotional mechanism for the observed higher well-being in older individuals. Furthermore, positive diversity may also be a dependable, objective route to assessing the positivity effect in this population since self-reported positive thought content varied between the two studies.

### Limitations

We attest this study has some limitations. Our samples were mostly White, and college educated, which may not generalize across other races/ethnicities or other educational attainments. We did not use a social desirability scale to determine whether participants’ answers to the ecological validity questions (i.e., censorship, similarity to daily life, and positive/negative thought reporting) were sincere. However, several features of the transcribed content, such as the presence of self-disclosure, self- or other-focused criticism, and cursing, converge to suggest that participants’ verbalized resting-state thoughts were largely sincere. Although it would have been ideal to acquire larger study samples, when aggregating the data and conducting a post hoc sensitivity analysis, we show that multiple regression with 96 participants across two study groups would be sensitive to effects of Cohen’s *f*^2^ = .083 with 80% power (alpha = .05). Future work could more directly examine the role of positive diversity in older adults’ well-being, including using causal manipulations where possible.

### Supplementary Information

Below is the link to the electronic supplementary material.

Supplementary file1 (DOCX 322 KB)

## Additional Information

### Data Availability

All data and scripts are available for download on the OSF repository: https://osf.io/gc3dm/.
