# Supplementary material for: Speaking Well and Feeling Good: Age-Related Differences in the Affective Language of Resting State Thought
Source: Affect Sci. 2024 Jun 24;5(2):141–59. doi: 10.1007/s42761-024-00239-z (PMC11264499; doi:10.1007/s42761-024-00239-z)
Supplement: Supplementary file 1 — Supplementary file1 (DOCX 322 KB) [file 42761_2024_239_MOESM1_ESM.docx]

**Speaking Well and Feeling Good: Age-related Differences in the Affective Language of Resting State Thought**

**(Supplemental Material)**

**SUPPLEMENTAL METHODS**

**Think Aloud Paradigm Instructions**

Study 1

“While in the scanner we are going to ask you to perform a type of ‘stream of consciousness’ task that will consist of you simply voicing out loud whatever comes to your mind for 7 minutes. At times your attention might be oriented outwards towards the sights and sounds of your external environment. At other times, you may be thinking about a particular topic, or your attention might be oriented towards your feelings, bodily sensations, and emotions. Regardless of what comes to mind at different moments in time, we would like you to continuously relay that information out loud. Please know that no one in the scanning room will actually be able to hear what you say. The person that transcribes your audio will not know your name and will not be the experimenter (i.e. Me) that was present during your experimental session. In summary, we ask that you remain awake, stay as still as possible, and simply voice out loud whatever you are experiencing. Any questions?”

Study 2 (Think-Aloud adaptation to virtual environment)

*“*We would like you to perform a type of ‘think aloud’ task that will consist of you simply voicing out loud whatever comes to your mind for 10 minutes. You’ll be asked to simply sit here in this room for 10 minutes and speak aloud your thoughts continuously as they are occurring. During this time, you may notice that at times, your attention might be oriented outwards towards the sights and sounds of your external environment. At other times, you may be thinking internally, about a particular topic, or your attention might be oriented towards your feelings, bodily sensations, and emotions. Regardless of what comes to mind at different moments in time, we would like you to continuously voice out loud what’s on your mind at that moment in time. Please know that no one in the testing area will actually be able to hear what you say. The person that transcribes your audio will not know your name and will not be the experimenter (i.e. Me) that was present during your experimental session. In summary, we ask that you remain awake and simply voice out loud whatever you are experiencing. Any questions?”

*Transcription Procedure:*

Nonfluencies – you should be transcribing all non-fluencies. LIWC recognizes the following non-fluencies: **er, hmm, sigh, uh, uhm, um**. If the sound the person makes doesn’t fit solidly into one of these options (e.g., ohhh), just choose the one that’s closest (e.g., uhhh).

In the case of a stutter, it is coded like a non-fluency using **um**. For example “Tr-tr-training is hard”, you would transcribe **um um training is hard** so that LIWC can recognize the two non-fluencies that are present in the stutter (i.e., it wouldn’t be able to recognize “tr-tr-“ as a nonfluency).

Fillers – fillers are the meaningless words we interject into sentences. You can tell it’s a filler if you can remove the word and the sentence is still grammatically correct and makes sense. As fillers are actually words (unlike non-fluencies) we need to make sure that LIWC doesn’t count them the same way as it would count regular words. We manipulate fillers so that LIWC recognizes them. They include: **youknow, Imean, Idontknow, ohwell, yaknow, youknow**.

Although LIWC can recognize those standard fillers, it can’t capture all the idiosyncrasies of people’s language. Thus, the tag **rr** can be used at the beginning of any word to denote that it’s a filler. For example, **like** is often used as a filler so to denote that it’s a filler (rather than someone saying they like something) we write **rrlike**. **Well** may also be used as a filler and would be written as **rrwell**. Please reserve rr for single words that are only being used as fillers. Some words have multiple definitions, and even if the word is not being used for its most common definition, LIWC can still capture secondary or tertiary definitions.

IMPORTANT: **Like** and **well** are not automatically fillers but if you can take them out of the sentence and it is still grammatically correct and makes sense, then it’s likely a filler.

*Assessing SES:*

We assessed subjective social status based on the MacArthur Scale of Subjective Social Status. The prompt is: "Imagine a ladder with 10 steps. At the top of the ladder are the people who are the best-off – those who have the most money, the most education, and the most respected jobs. At the bottom are the people who are the worst-off – those who have the least money, least education, and the least respected jobs (or no job). The higher up you are on this ladder, the closer you are to the people at the very top; the lower you are, the closer you are to the people at the very bottom.” It is rated from 1 (lowest) to 10 (highest).

**SUPPLEMENTAL RESULTS:**

***Supplementary Tables***

**Table 1**

**Table 2**

**Table 3**

***Study 2 relationships between vocabulary knowledge and primary diversity metrics***


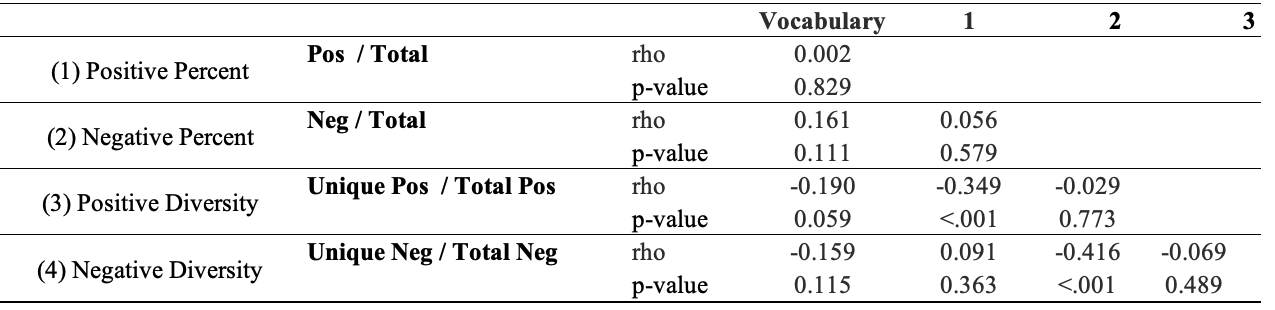


**Table 4**


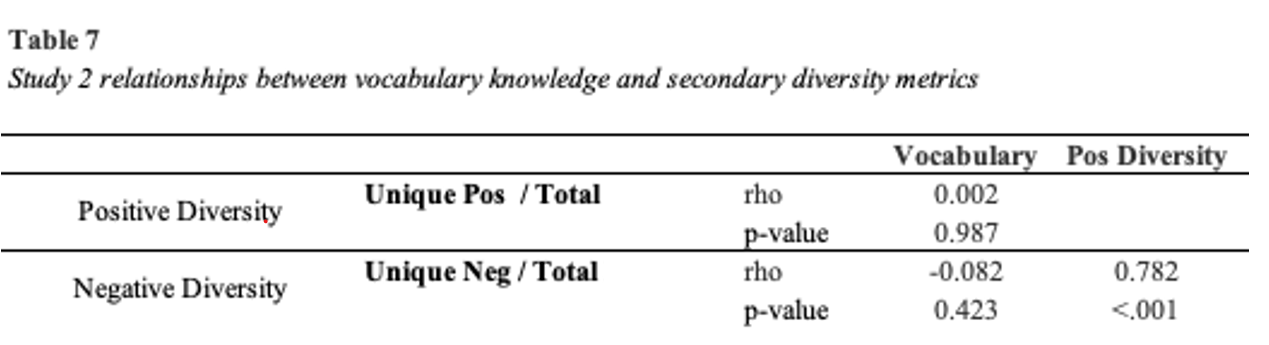


**Secondary Diversity Metrics Results**

Exploratory secondary metrics of diversity were computed that did not control for differences across participants in the number of positive or negative words. These metrics used the identical formula employed by the Vocabulate software (Vine et al., 2020) and differed in their denominator from our main metrics; they were computed simply for readers’ reference to this previous study. The results are presented below.

**Affective Linguistic Diversity (Study 1)**

Older adults exhibited more positive diversity compared to younger adults (ratio of unique positive words to total words; older adults: *Mdn*=.122, *M*=.118; young adults: *Mdn*=.095, *M*=.108; *U*=187, *p*=.041, *RBC*=.346), but did not differ in the ratio of unique negative words to total words (older adults: *Mdn*=.019, *M*=.020; young adults: *Mdn*=.018, *M*=.019; *U*=258, *p*=.573, *RBC*=.097).

**Affective Linguistic Diversity (Study 2)**

Older adults did not differ in the ratio of positive unique words to total words compared to younger adults (Older adults: *Mdn*=.094, *M*=.109; young adults *Mdn*=.082, *M*=.095), *U*=1169, *p*=.320, *RBC*=.114, or in the ratio unique negative words to total words (Older adults: *Mdn*=.015, *M*=.019; Young adults: *Mdn*=.015, *M*=.016), *U*=1238, *p*=.590, *RBC*=.062.

**Aggregated Study Results**

Older adults did not exhibit higher positive diversity when using the formula from Vine et al. 2020 (OA: *M*=.090, *Mdn*=-.112, YA:*M*=-.110, *Mdn*=-.308, *U*=956, *p*=.167, *RBC*=.118), nor negative Diversity (OA: *M*=.126, *Mdn*=.078, YA:*M*=-.149, *Mdn*=-.000, *U*=984, *p*=.231, *RBC*=.118). Similarly, positive diversity was not related to wellbeing with the secondary quantification of positive diversity (unique positive words/total words), Spearman’s rho (94) =.054, *p*=.65.
